# Self-Attention MHDNet: A Novel Deep Learning Model for the Detection of R-Peaks in the Electrocardiogram Signals Corrupted with Magnetohydrodynamic Effect

**DOI:** 10.3390/bioengineering10050542

**Published:** 2023-04-28

**Authors:** Moajjem Hossain Chowdhury, Muhammad E. H. Chowdhury, Muhammad Salman Khan, Md Asad Ullah, Sakib Mahmud, Amith Khandakar, Alvee Hassan, Anas M. Tahir, Anwarul Hasan

**Affiliations:** 1 Department of Electrical, Electronic and System Engineering, Universiti Kebangsaan Malaysia, Bangi 43600, Malaysia; 2 Department of Electrical Engineering, Qatar University, Doha 2713, Qatar; 3 Department of Mechanical and Industrial Engineering, Qatar University, Doha 2713, Qatar; 4 Department of Biomedical Engineering, Military Institute of Science and Technology, Mirpur Cantonment, Dhaka 1216, Bangladesh

**Keywords:** magnetohydrodynamic (MHD) effect, magnetic resonance imaging (MRI), electrocardiogram (ECG), operational neural networks (ONN), R-peak detection, feature pyramid network (FPN)

## Abstract

Magnetic resonance imaging (MRI) is commonly used in medical diagnosis and minimally invasive image-guided operations. During an MRI scan, the patient’s electrocardiogram (ECG) may be required for either gating or patient monitoring. However, the challenging environment of an MRI scanner, with its several types of magnetic fields, creates significant distortions of the collected ECG data due to the Magnetohydrodynamic (MHD) effect. These changes can be seen as irregular heartbeats. These distortions and abnormalities hamper the detection of QRS complexes, and a more in-depth diagnosis based on the ECG. This study aims to reliably detect R-peaks in the ECG waveforms in 3 Tesla (T) and 7T magnetic fields. A novel model, Self-Attention MHDNet, is proposed to detect R peaks from the MHD corrupted ECG signal through 1D-segmentation. The proposed model achieves a recall and precision of 99.83% and 99.68%, respectively, for the ECG data acquired in a 3T setting, while 99.87% and 99.78%, respectively, in a 7T setting. This model can thus be used in accurately gating the trigger pulse for the cardiovascular functional MRI.

## 1. Introduction

The use of functional magnetic resonance imaging (fMRI) has been proven to be an excellent method for evaluating the functional condition of the heart [1]. Other imaging methods, such as echocardiography, cardiac computed tomography (CT), and nuclear medicine, are complemented by cardiovascular fMRI, which plays an important role in the diagnosis and treatment of cardiovascular diseases, as well as in research [2]. It is also standard for assessing cardiac structure and function [3]. fMRI can also be used to obtain functional information regarding vascular blood flow. Along with being a valuable tool for diagnosis, fMRI is also used in surgical planning of complex congenital heart diseases [4]. Procedures such as taking biopsies, tumor therapies, and electrophysiological studies benefit from fMRI.

A prerequisite for cardiovascular fMRI is the adequate synchronization of image acquisition with the cardiac cycle [5]. For that, the use of an accurate triggering pulse is required to permit the successive acquisition of imaging sequences in line with the phase of the heart. It is possible to generate the triggering pulse using a variety of methods, including photoplethysmography signals [6], a Doppler ultrasound [5]-based method, and an optical-based sensor [7]. However, the most common and straightforward way to accomplish this goal is to record an electrocardiogram (ECG) in parallel with MRI and use the location of the R-peak on the ECG to trigger the acquisition [8,9]. Therefore, good synchronization is necessary for collecting precise data in fMRI, which is highly dependent on the precise detection of R-peaks from the ECG signal. Additionally, concurrently recording an ECG with an MRI scan can also be used for diagnostic purposes. ECG is an essential tool for evaluating cardiovascular function and frequently appears in clinical practice. Because it might be challenging to visually monitor or converse with the patient while inside the MRI scanner, the attending physician or clinical staff must rely on the patient’s vital data to make a diagnosis. MRI patients, especially those in critical or unstable condition, need to be adequately monitored during the procedure [10]. For example, patients coming from an intensive care unit (ICU) or patients under anesthesia need proper monitoring. A diagnostically useful surface ECG is unavoidable for MRI-guided electrophysiological (EP) operations, which are sparingly carried out in modern day practice, but have the potential to become more significant in the foreseeable future [11,12].

**Figure 1:**
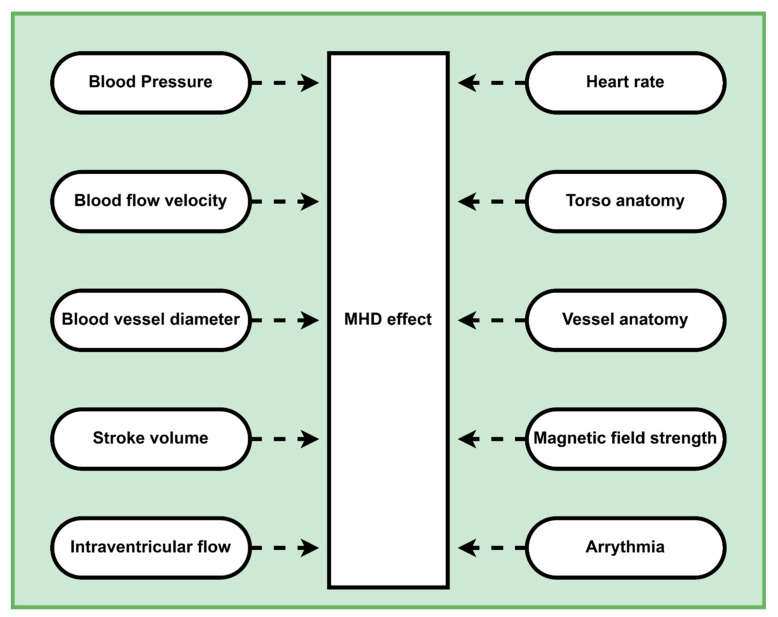
Physiological and technical parameters that influence the MHD effect.

However, the imaging setup (high static magnetic field, gradient switching, radio frequency (RF) pulse) of MRI greatly undermines the synchronization process by heavily distorting the recorded ECG signal. A high magnetic field mainly distorts the ECG signal, known as the Magnetohydrodynamic (MHD) effect [13,14]. Blood, consisting of charged ions, is subjected to a continuous flow due to the pumping activity of the heart. When a subject is placed under a magnetic field, the magnetic field exerts a force on the dynamic ions of the blood [14]. This force is known as Lorentz force, and it acts perpendicular to the direction of the applied magnetic field and the direction of the blood flow. Due to this, ions distribute themselves in the periphery of blood vessels and produce an electric voltage. The induced potential due to the MHD effect superimposes the recorded ECG signal and alters the waveform of the recorded signal. Many other parameters influence the MHD effect, as summarized in Figure 1. In the distorted ECG signals, the amplitude of the T wave eclipses that of the R-peak and makes it difficult to detect the location of R peaks [15]. Similarly, in most cases, due to the superposition of the ECG and MHD signals, a detailed and reliable morphological analysis of the ECG (e.g., the P wave, ST segment, or the T wave) during MRI exams is not possible [16,17,18,19,20]. Another challenge is the detection of the QRS complex. Depending on the characteristics of the MHD signal, QRS detection might be hampered [21].

Numerous studies have examined the effects of various noise sources, including the MHD effect and artifacts, on ECG signals. In addition, abnormal ECG signals, such as arrhythmia and premature ventricular contraction [22], have been investigated. There have also been studies conducted that deal with separating fetal ECG from mother ECG [23]. These studies were conducted in various experimental settings, and multiple algorithms have been proposed for detecting R-peaks, generating trigger signals, and classifying normal and abnormal ECG signals. During the earlier stages of working with MHD-affected ECG signals, extraction of referenced ECG signals from the distorted signal was predominant. In the extraction of referenced ECG signals, various filtering techniques were incorporated, such as Independent Component Analysis (ICA) [9], Wilcoxon filter [24], Least mean squares (LMS) adaptive filtering [25], as well as other adaptive filtering methods.

Numerous studies have been conducted on the detection of R-peaks in non-MHD corrupted ECG signals. One of the most popular methods is the Pan-Tomkins algorithm [26]. The algorithm uses filtering, differentiation, squaring, and thresholding to detect the R-peaks. Adaptive filtering and template matching have been used to further improve the detection performance. Continuous wavelet transform with selective scale [27] and Shannon’s energy [28] have also been used to detect R-peak locations, while discrete wavelet transform was used in [29]. In [30], the authors used sorting and thresholding squared double difference signals from ECG data to estimate R-peak locations. Another study conducted by Mabrouki et al. [30] consisted of cleverly combining Hilbert transform and empirical mode decomposition. While, in [31], a novel empirical mode decomposition algorithm was used, called ensemble empirical mode decomposition with the adaptive noise (CEEMDAN). The algorithm addresses the issue of ‘mode-mixing’ and different realizations of signal with Gaussian white noise. The method uses modes 2–5 from CEEMDAN to detect the R-peak locations.

In terms of R-peak detection in MHD corrupted signals, Vectorcardiogram (VCG) [32,33,34,35] had been extensively used along with the signal processing techniques before the adoption of decomposition techniques. The extraction of the R-peaks reference vector from the ECG signal recorded outside of an MRI scanner was a prerequisite for this method. The location of R-peaks in MHD affected the ECG signal and was determined using projections of the VCG signal across the reference R-peak’s directions [32], where the reference R-peak is the extracted R-peak vector from the ECG signal taken outside of the MRI scanner. Euclidean, as well as cosine [32], directions were incorporated with the projection for better accuracy. Derivative-based methods [33] have also been studied in various experiments. Krug et al. [9] used Independent Component Analysis (ICA) to diminish the effects of MHD in ECG waveform, and R-peaks were then detected. Twelve-lead ECG data were processed using ICA after being captured in a 7T MR scanner. To locate the dominant independent component (IC) in the ECG signal, an automated source identification approach was presented. Once the IC was selected, it could be utilized for R-peak detection. The decomposition-based technique supplanted other signal processing-based techniques with the emergence of wavelet decomposition and wavelet transform to detect R-peaks [36], or for signal processing to extract the reference ECG signal [36,37] with greater accuracy. With the improvements in computation power, both machine learning associated with signal processing [38,39,40] and deep-learning-based approaches [41,42,43] have become ubiquitous and have been intensively used in the detection of R-peaks/beat detection in noisy and abnormal ECG signals.

The motivation of this study is to devise a robust R-peak detection system for MHD corrupted ECG signals using a deep learning technique that seemed promising for highly corrupted Holter ECG datasets [44]. The authors believe that the inherent adaptability of deep learning models will help handle the noisy nature of the data. To this end, this work aims to contribute to the field by:

- Proposing a novel deep learning model, Self-Attention MHDNet, which can accurately detect R-peaks by approaching the problem as a segmentation problem.
- Assessing the performance of the model on ECG data collected from both 3T and 7T MRI machines.
- Pioneering the use of deep learning models for R-peak detection in MHD corrupted ECG signals.
- Demonstrating that three-channel ECG signals are sufficient for detecting R-peaks in multi-channel ECG signals.

The manuscript is organized as follows: In Section 2, the methodology employed in this study is expounded, encompassing a concise depiction of the dataset, the problem formulation, the network architecture, the training methodology, and the evaluation metrics. The outcomes of the ablation study, the evaluation of the model, and a comparison with the current literature are presented in Section 3. Finally, the manuscript is concluded with Section 4.

## 2. Materials and Methods

In this section, the experimental setup, problem formulation, dataset description, pre-processing steps, and the proposed deep neural network architecture along with training methodology for detecting R-peaks in MHD-corrupted ECG signals are discussed. Finally, the various metrics used for evaluating the model are described.

**Figure 2:**
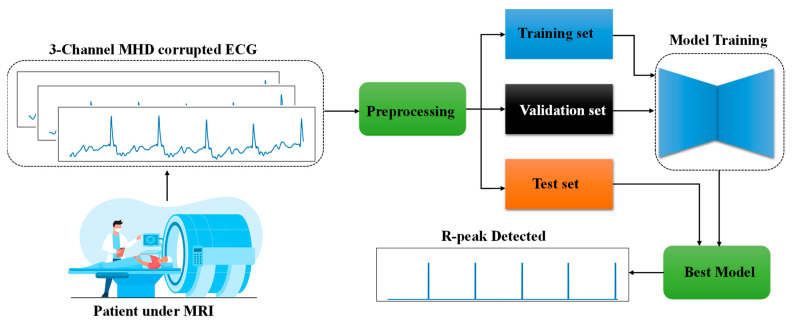
Detection of R peaks in three-channel ECG waveforms collected from patients under MRI.

The process of detecting R-peaks from patients inside MRI has been summarized in Figure 2. Patients were scanned using an MRI scanner while their ECG signals were recorded. Due to the presence of MRI magnetic fields, the ECG signals were corrupted by the MHD effect. The corrupted ECG signals were first preprocessed, then they were split into training, validation, and test sets. The training set was used for training the proposed model, while the validation set was used to choose the best model. The resulting best-trained model was then used on test data to detect R-peaks.

### 2.1. R-peak Detection as av Segmentation Problem

**Figure 3:**
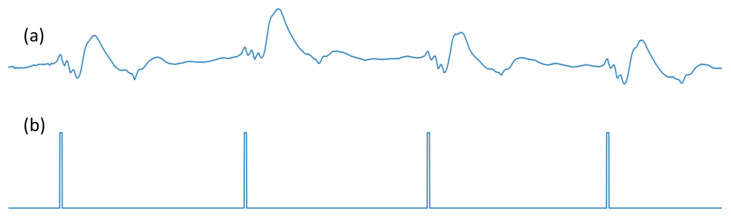
R-peak detection as a segmentation problem. Here, (**a**) depicts Channel 1 or Lead I of corrupted ECG, and (**b**) depicts the R-peaks as a pulse train.

Segmentation is a popular method in the biomedical image and signals domain for its ability to isolate the regions of interest (ROIs) [45]. Most segmentation models are variants of the UNet model [46], which consist of an encoder and a decoder. The 2D segmentation models take an image as input and produce a mask where the region of interest is depicted as 1, while the background is depicted as 0. This methodology can be adopted for detecting R-peaks in ECG signals, based on our previous work [44]. In this case, the region of interest is the R-peak location. The R-peak locations are manually annotated by expert physicians. The R-peaks are modeled as a rectangular pulse with a height of 1.0 and a length of 13 samples (roughly 12.7 ms) [44]. A corrupted ECG and its corresponding R-peaks as pulses are shown in Figure 3. The signals are ‘widely’ plotted so that the width of the pulse train can be easily seen. In this work, the proposed model will map a corrupted three-channel ECG signal to a pulse train, where the pulses refer to the R-peak locations.

### 2.2. Dataset Description

The dataset used here is taken from Krug et al. [47], as they made the dataset public in “PhysioNet” [48,49]. The motivation behind the collection of this dataset was to carry out further research on analyzing the ECG signals corrupted by the strong static magnetic fields generated by MRI machines. The dataset contained 53 records from 29 subjects. The data is annotated by either Physicians or ECG experts. In this work, ECG data corrupted by 3T (23 subjects) and 7T (5 subjects) magnetic fields are used, as the number of subjects for 1T (only 1 subject) is insufficient for training deep learning models. Moreover, in this dataset, the number of ECG channels is not uniform for all subjects. Some subjects have twelve channels, while the others have only three channels (Lead I, II, and III). Therefore, for both 3T and 7T machines, three channel ECG signals have been used. The dataset was sampled at 1024 Hz and segments of 4 s duration were applied as input to the deep learning models.

### 2.3. Preprocessing

**Figure 4:**
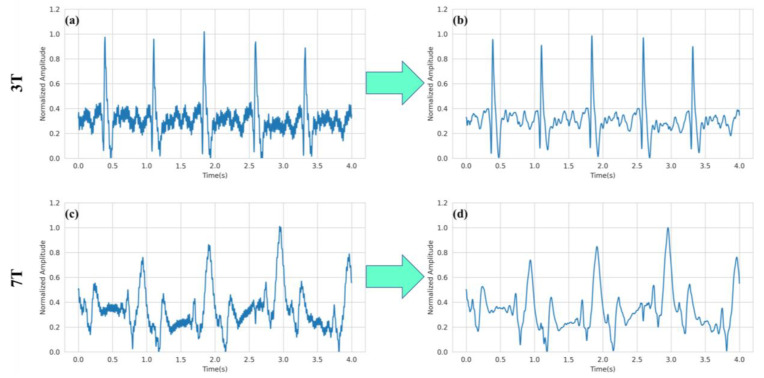
Result of preprocessing on MHD corrupted ECG signals. The preprocessing steps convert the raw ECG signal corrupted by a 3T magnetic field in (**a**) to obtain the clean signal in (**b**). The same steps were also taken to filter raw ECG signal corrupted by 7T magnetic field in (**c**) to obtain the clean signal in (**d**).

The MHD corrupted ECG signals were filtered with a bandpass filter of 0.05 Hz to 100 Hz bandwidth. After the bandpass filtering, a notch filter or narrowband band stop filter with a central rejection frequency of 50 Hz was applied to clean power line distortions. Figure 4 depicts the effect of preprocessing steps on raw ECG signals. The waveforms in Figure 4a,c show ECG signals corrupted by magnetic fields of 3T and 7T, respectively. The aforementioned preprocessing steps were applied to both signals. The result of the preprocessing steps on signals in Figure 4a,c are shown in Figure 4b,d, respectively.

### 2.4. Model Architecture

In this work, a novel architecture is proposed for detecting R-peaks via a segmentation model, named Self-Attention MHDNet. The network utilizes the concepts of Self-Organizing Operational Neural Networks (Self-ONN) [50,51,52,53,54], Feature Pyramid Networks (FPN) [55], and Attention mechanism. This subsection explains the Self-ONN layers, the actual network, and how the Attention mechanism works.

#### 2.4.1. Self-ONN

This work proposes a new architecture using Self-ONN [50,51,52,53,54] for R-peak detection. Operational Neural Networks (ONNs) employ generative neurons rather than homogeneous linear approximations used by Convolutional Neural Networks (CNNs) [50,51,54]. ONNs are conceptual expansions of the neural network class, Generative Operational Perceptrons (GoPs) [51]. Self-ONNs are an efficient version of ONN, where the operators are no longer selected from a library of operators. Because genuine neurons execute a wide range of neurochemical processes, these ONNs and Self-ONNs emulate them by simulating numerous synaptic connections and operations in the deep learning layer. For an input feature $x^{n-1}$, of $n^{th}$ neuron, the approximation function $f\left(x\right)$ can be formulated by using Equations (1)–(3).

(1) $$f\left(x\right)=f\left(x_{0}\right)+\frac{\;f\prime \left(x_{0}\right)}{1!}\left(x-x_{0}\right)+\frac{\;f\prime \prime \left(x_{0}\right)}{2!}{\left(x-x_{0}\right)}^{2}+\cdots +\frac{f^{q}\left(x_{0}\right)}{q!}{\left(x-x_{0}\right)}^{q}$$

(2) $$f\left(x\right)=f\left(0\right)+\frac{\;f\prime \left(x_{0}\right)}{1!}\left(x\right)+\frac{\;f\prime \prime \left(x_{0}\right)}{2!}{\left(x\right)}^{2}+\cdots +\frac{f^{q}\left(x_{0}\right)}{q!}{\left(x\right)}^{q}$$

(3) $$f\left(x\right)=b+\omega_{1}\left(x\right)+\omega_{2}{\left(x\right)}^{2}+\cdots +\omega_{q}{\left(x\right)}^{q}$$

This approximation is derived with the help of the Taylor Series approximation. As shown in Equation (3), b is the bias that is formulated from the Taylor series approximation on x→0. For Self-ONN layers, tanh activation, instead of ReLU, is used so that the approximation is bound between −1 and 1. Several studies have shown that Self-ONN-based model designs outperform CNN-based architecture [53,54,56]. Previous work has also studied how Self-ONN compares in R-peak detection for Holter ECG signals [44]. Hence, it is important to examine the effectiveness of a deep Self-ONN model for R-peak detection in MHD-affected ECG.

#### 2.4.2. Self-Attention MHDNet

**Figure 5:**
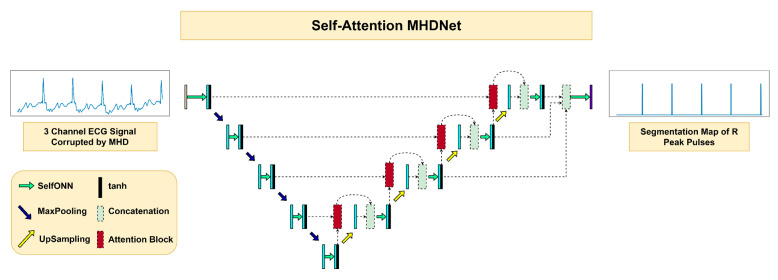
Network architecture of the proposed Self-Attention MHDNet.

The model, as shown in Figure 5, contains four layers in the encoder section and four layers in the decoder section with a bottleneck in between. Each Self-ONN layer (q = 3) is followed by an instance norm and tanh activation. The architecture requires the signal length to decrease as we go deeper and for it to increase as we go ‘upwards’ from the deeper layers. For decreasing the signal lengths, we use a max pooling layer, which decreases the length by a factor of 2. For increasing the signal lengths, we use an upsampling layer, which increases the length by a factor of 2. As a result, the signal length is halved as we go deeper. The first layer of the encoder contains only 16 filters. The number of filters doubles as we go deeper into the network, with the bottleneck having 256 filters. The kernel size is kept the same throughout the model with a size of 11.

In place of a normal skip connection between the encoder layer and the decoder layer, an attention block is placed in between to focus on the relevant parts of the signal. The attention block takes in the encoder signal from the $i^{th}$ layer and the decoder signal from the $i+1^{th}$ layer. The attention-guided signal is then concatenated with the decoder signal from the $i^{th}$ layer.

In a normal encoder-decoder architecture, the feature map of the final layer is passed through an ONN layer to obtain the final segmentation mask. However, taking inspiration from FPN, a feature concatenation approach is taken. Feature maps from the first, second, and third decoder layers are concatenated together. Deeper layer feature maps are interpolated to match the shape of the feature maps in the output layer (layer 1). The resulting feature map is then passed through an ONN layer of kernel size 1 that produces the final output.

#### 2.4.3. Attention Mechanism

**Figure 6:**
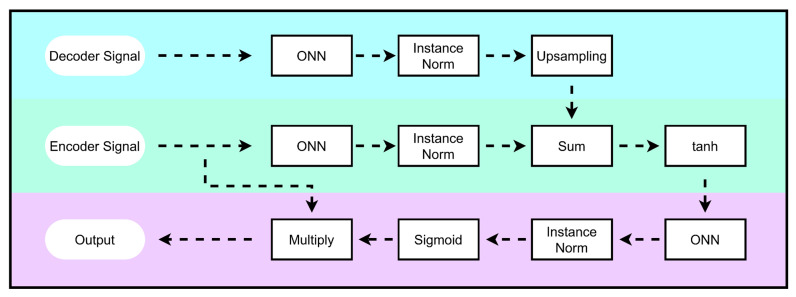
Attention block. The Decoder signal from the (*i* + 1)*^th^* layer and encoder signal from the *i^th^* layer are taken as inputs into the attention block.

The attention mechanism helps in focusing the features of the model into maximum relevancy. Figure 6 demonstrates the attention mechanism in detail. The attention block takes in two signals viz. the encoder signal (from $i^{th}$ layer) and the decoder signal (from $i+1^{th}$ layer). Both the encoder and decoder go through an ONN layer of kernel size 1 followed by instance normalization. The ONN layer has the same number of filters as the number of channels in the signal. The decoder signal is then upsampled by a factor of 2, and then added to the encoder signal in a summation operation. The resulting signal is introduced to some non-linearity in the form of tanh activation.

The signal is then passed through an ONN layer of 1 filter with kernel size 1, which is later normalized via instance normalization. We now have a vector that is the same length as the encoder signal. Another non-linearity is introduced in the form of sigmoid activation so that the vector is between 0 and 1. The vector is then multiplied elementwise with the encoder signal. The product is then given as the output of the attention block.

### 2.5. Training Methodology

The dataset is split into five folds, where three folds are used for training, one fold for validation, and one fold for testing. The split is carried out in such a way that there is no leaking of the same subject’s data between the folds. The results reported in this study were calculated by averaging over all five folds. The training data is split into segments of 4-s duration with a 75% overlap. The process is the same for both 3T and 7T. This ensures that there is enough data for the training phase. This resulted in 2809 segments for 7T data and 9171 segments for 3T data. A batch size of 128 is used to train the model for 100 epochs. The Adam optimizer is used to optimize the cross-entropy loss with a learning rate of 1 × 10^−3^.

### 2.6. Evaluation Criteria

The trained models were quantitatively evaluated in two categories: segmentation and R-peak detection. Intersection over Union (IoU) and Dice Similarity Coefficient (DSC) are computed to robustly quantify the performance of the network in 1D segmentation mask generation. IoU and DSC are calculated using Equations (4) and (5), respectively. Here, TP, FN, and FP refer to true positive, false negative, and false positive, respectively, in terms of the segmented 1D waveform data points.

(4) $$\mathrm{IOU}=\frac{\mathrm{TP}}{\mathrm{TP}+\mathrm{FN}+\mathrm{FP}}\times 100\%$$

(5) $$\mathrm{DSC}=\frac{2\times \mathrm{TP}}{2\times \mathrm{TP}+\mathrm{FN}+\mathrm{FP}}\times 100\%$$

For R-peak detection, three metrics are employed viz. recall, precision, and F1-score, as shown in Equations (6)–(8), respectively. It is also essential to note that the number of true positives, false positives, and false negatives was obtained within 70 milliseconds of the true peak location [44]. For R-peak detection, TP, FP, and FN refer to instances where the R-peak is properly detected, falsely detected, or not detected, respectively.

(6) $$\mathrm{Recall}=\frac{\mathrm{TP}}{\mathrm{TP}+\mathrm{FN}}\times 100\%$$

(7) $$\mathrm{Precision}=\frac{\mathrm{TP}}{\mathrm{TP}+\mathrm{FP}}\times 100\%$$

(8) $$F1-\mathrm{score}=\frac{\mathrm{TP}}{\mathrm{TP}+\frac{1}{2}\left(\mathrm{FP}+\mathrm{FN}\right)}\times 100\%$$

For easy readability, the five metrics were converted from ratios to percentages.

## 3. Results and Discussion

In this section, we present the experimental outcomes of the study with brief discussions on each. Firstly, the authors conduct an ablation study regarding the importance of various blocks that make up the proposed network. The authors then analyze the R-peak detection capability of the model in 3T and 7T data. Finally, the performance of the proposed network is compared to the current literature in R-peak detection of MHD corrupted signals. The models were newly trained for both 3T and 7T data.

### 3.1. Ablation Study

**Figure 7:**
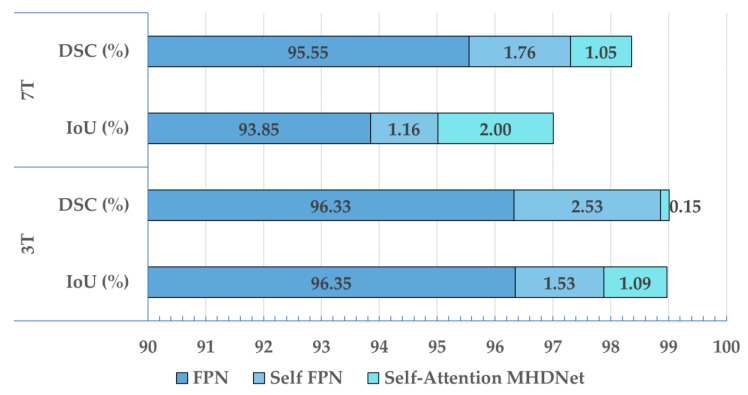
Outcomes of the ablation study for the effect of Self-ONN layers and attention mechanism on the FPN model. Note that in this stacked bar plot, IoU and DSC for FPN are shown as the base performance, while the Self-FPN and Self-Attention MHDNet outcomes are shown as improvements.

To verify the effect of the various modules on the proposed model, an ablation study is conducted where the effect of Self-ONN layers and attention mechanism is studied. In each experiment, the model is newly trained following the structure discussed in Section 2.5. The results of the ablation study are shown in Table 1 and Figure 7. An FPN architecture (using CNN instead of Self-ONN) is used as the baseline model, while all other parameters are kept the same. The baseline model performs well with IoU and DSC of 96.35% and 96.33% for 3T, and 93.85% and 95.55% for 7T. Changing CNN layers to Self-ONN layers results in IoU and DSC increasing to 97.88% and 98.36% for 3T, and 95.01% and 97.31% for 7T. For 3T, it shows an improvement of 1.53% and 2.53% in terms of IoU and DSC. On the other hand, 7T shows an improvement of 1.16% and 1.76% in terms of IoU and DSC.

Adding an attention mechanism pushed the model’s performance to an even higher level. The IoU increased from 97.88% to 98.97% (3T) and from 95.01% to 97.01% (7T) when compared to the Self-FPN model. The DSC increased from 98.86% to 99.01% (3T) and from 97.31% to 98.36% (7T) for the same situation. This addition caused the most improvement in IoU for 7T, as it increased by 2.00%. Thus, replacing CNN with Self-ONN layers and then adding an attention mechanism appears to be a promising method for detecting R-peaks in MHD-affected ECG.

### 3.2. R-peak Detection Analysis

The ablation study proved that Self-Attention MHDNet outperforms all other variations of FPN in segmenting the R-peak pulse train by a significant margin. Hence, the performances of R-peak detection are computed on the results of that model where the Self-Attention MHDNet model has been separately trained for both 3T and 7T data. The previously discussed evaluation metrics are shown in Table 2. It can be observed that recall, precision, and F1-score for both settings are above 99%. Furthermore, the F1-score is used as the main metric as it is a harmonic sum of recall and precision. Considering that, even though both models performed very well, the performance in the 7T setting marginally outperformed the model in the 3T setting.

**Figure 8:**
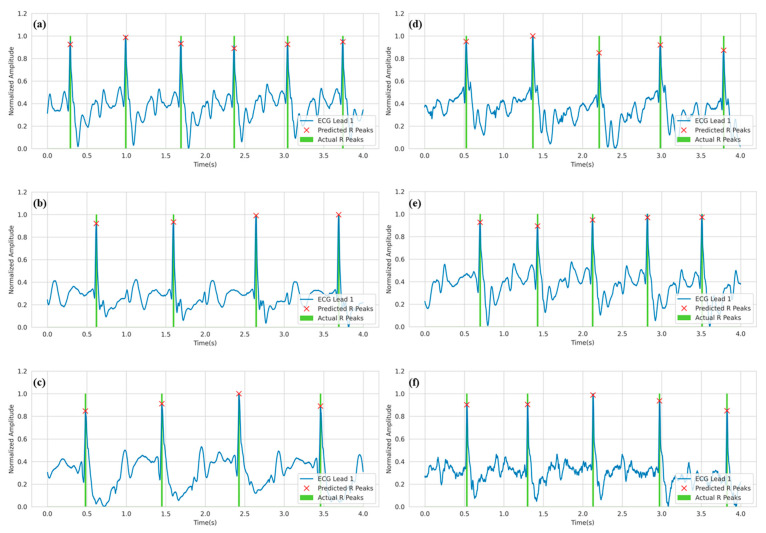
R-peak detection for ECG Lead I in the 3T setting where (**a**–**f**) refer to various samples from the test set. Green spikes denote the ground truth R peaks annotated by clinical experts, while the red crosses denote the predicted R-peaks.

For qualitative evaluation, Figure 8 depicts the prediction of R-peaks from three-channel ECG waveforms under the 3T setting. Only channel 1 or lead I of the ECG is visualized. The green shaded area shows the location of the actual R-peak. In the 3T setting, the R-peaks correspond to the highest peaks in the signal. This is because the signal is not distorted to a high degree due to the MHD effect. As a result, the model can easily learn the patterns for R-peak locations compared to the 7T setting, which will be described later.

**Figure 9:**
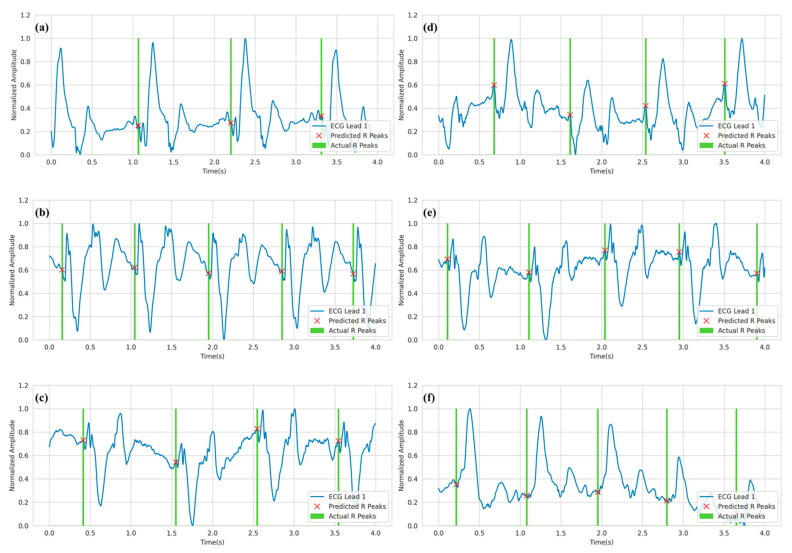
R-peak detection for ECG Lead I data in 7T setting where (**a–f**) refer to various samples from the test set. Green spikes denote the ground truth R peaks as annotated by clinical experts. On the other hand, the red cross denotes the predicted R-peaks.

Similar to the 3T setting, Figure 9 depicts the predicted R-peaks from three-channel ECG waveforms under a 7T magnetic field. The first observation from the figure is that the ECG waveforms are distorted to a very high degree. While in some cases, the R-peaks are in a trough before the largest peak (as in Figure 9f), in other cases, the R-peaks are in a smaller peak before the largest peak (as in Figure 9d). Despite that, the model was able to reliably predict the R-peaks in the corrupted ECG signals.

**Figure 10:**
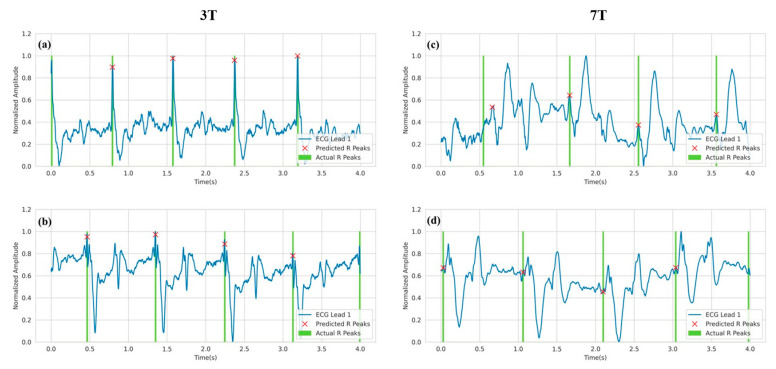
Analysis of missed R-peaks in 3T setting (**a**,**b**) and 7T setting (**c**,**d**).

As shown in Table 1 and Table 2, the model is not 100% accurate. There are some missing cases. Figure 10 depicts some signals where the model failed to detect some R-peaks in the signal. It is important to note that the model correctly identified most of the R-peaks in the waveform. In Figure 10, two types of errors are noticed. In Figure 10a,b,d, the model missed the R-peaks when it was almost out of bounds. That was the most common error in this work. The other error can be seen in Figure 10c, where the model missed the R-peak by roughly 0.2 s. It is important to notice that the second type of error only occurred for ECG under 7T settings.

### 3.3. Heart Rate Analysis

**Figure 11:**
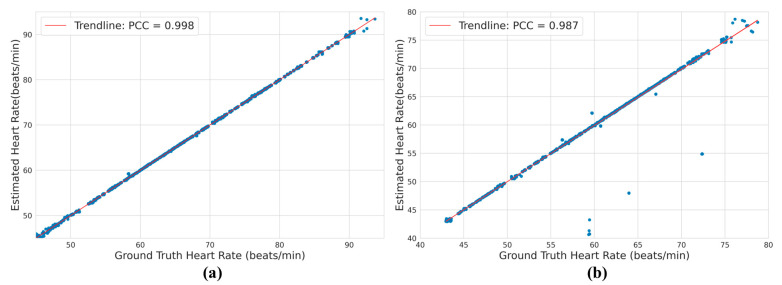
Analysis of heart rate prediction using a regression plot for ECG under a (**a**) 3T and a (**b**) 7T setting.

The proposed system is very accurate in predicting R-peaks in ECG waveforms. While R-peaks can be used for gating under an MRI machine, they can also be used to monitor the heart rate (HR). Abnormal heart rates often signify that the patient is under duress. To demonstrate the performance of the proposed system in heart rate estimation, the R-peaks were used to calculate the R-R intervals and, consequently, the heart rate. The actual heart rate and predicted heart rate were calculated from actual and predicted R-peaks, respectively. The heart rates were analyzed with the help of a regression plot, as shown in Figure 11. In a regression plot, the estimated heart rate is plotted against the actual heart rate. Then, a trendline is fitted through the data points. An ideal model will have a trendline with a gradient, i.e., a Pearson’s Correlation Coefficient (PCC) of 1. As seen in Figure 11, the trendline almost perfectly fits the data points for both 3T and 7T, and shows a very high correlation. Moreover, the heart rate estimation for the 3T setting has a PCC of 0.998 compared to the PCC of 0.987 for the 7T setting; which makes sense, as the 3T magnetic field distorts the signal less than the 7T magnetic field. Nevertheless, our proposed approach showed excellent performance in estimating heart rate.

**Figure 12:**
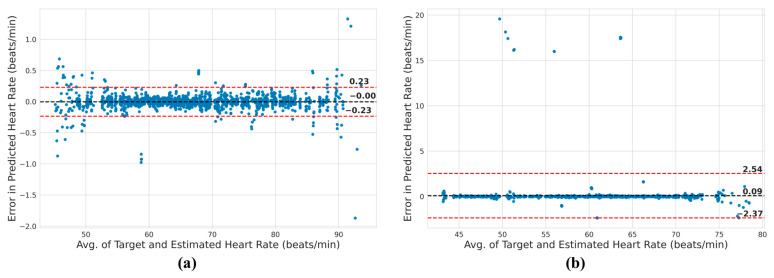
Heart rate estimation analysis using Bland-Altman plot for ECG under a (**a**) 3T and a (**b**) 7T setting. The black dotted line corresponds to the average value of the variances, whereas the red dotted lines depict the range within which 95% of the variances are expected to fall.

Further analysis of the estimated and actual heart rate was carried out using the Bland-Altman plot in Figure 12. In a Bland-Altman plot, the difference in estimated and actual heart rate is plotted against the average of the estimated and actual heart rate. The black dotted line represents the average of all the differences, while the red dotted lines represent the 95% confidence interval. Hence, in ideal conditions, the plot will contain all the data points that have a y-coordinate of 0. It will essentially be a horizontal line along the x-axis. In this case, Figure 12 shows that the spread of error in the prediction of heart rate is quite small. The 95% confidence intervals for 3T and 7T settings range from 0.23 beats per minute (bpm) to −0.23 bpm, and from 2.54 bpm to −2.37 bpm, respectively.

### 3.4. Comparison with Current Work

The results obtained from an experiment must be compared with the literature. However, it is very difficult to do so in tasks where the main resource, the dataset, is scarce. Hence, to ensure a fairer comparison, the performance of various methods that used a version of the dataset used in this work is reported in Table 3. Unfortunately, no literature could be found that used the exact data and no work was found that used 3T data of this dataset. The methods that have been reported were all implemented by Krug et al. [9]. Krug et al. first showed the performance of the methods M1, M2, M3, and M5 on the 7T data. M1 used an ECG lead and showed a respectable precision and recall of 89.40% and 87.10%.

However, in M2, when VCG was used, the performance jumped to 91.20% and 88.90% in precision and recall, respectively. When 3D VCG was used in M3, the results drastically dropped. The precision was less than 60% and the recall was 72%. M5 used an ICA-based method that had a precision of 87.50% and recall of 84.30%. Krug et al. chose to improve this ICA-based method and were able to obtain very good precision and recall of 99.10% and 99.20%, respectively. Our method, however, outperformed [9] this with a precision of 99.87% and a recall of 99.78%.

Despite accurately predicting the R-peaks in the ECG waveforms, which are corrupted under 3T and 7T magnetic fields, the model still needs to be tested for robustness in external datasets, which are not currently available. Another limitation is that the proposed model needs a separate model for 3T and 7T. It might not properly work in cases where the field strength will be different. In the future, the authors aim to create a model that will be able to robustly detect the R-peaks regardless of the degree of MHD effect on ECG. To ensure robustness, the model could be evaluated with external data. These studies would, however, require extensive data collection. This study is a step towards building a universal R-peak detector in MHD-corrupted ECG signals.

## 4. Conclusions

In the field of medical diagnosis and image-guided interventions, Magnetic Resonance Imaging (MRI) is a commonly used technique. An electrocardiogram (ECG) may be used to monitor the patient’s heart during an MRI scan to ensure cardiac gating, capture information at end diastole or end systole, or acquire partial images throughout the cardiac cycle and average these signals out over several heartbeats. However, the strong magnetic fields present in an MRI scanner can lead to significant distortions of the ECG data due to the Magnetohydrodynamic (MHD) effect. These distortions, which can cause abnormalities in the heartbeat pattern, make it challenging to detect QRS complexes and limit the diagnostic potential of ECG readings. Hence, in this work, a novel network, Self-Attention MHDNet, was proposed to detect R-peaks in the ECG waveforms collected inside an MRI scanner. This model formulates the detection process as a 1D-segmentation problem. In a 3 Tesla (T) environment, the proposed model attained precision and recall percentages of 99.83% and 99.68%, respectively. On the other hand, in a 7T environment, the model could obtain precision and recall rates of 99.87% and 99.78%, respectively. It was also shown that only three channels of ECG (Lead I, II, and III) are enough to accurately detect R-peaks in ECG signals distorted by the MHD effect. Furthermore, an ablation study was conducted, where it was found that the addition of Self-ONN layers and the attention mechanism improved the segmentation capabilities of the model. Therefore, the proposed approach could be used to robustly detect the locations of the R-peaks in the MHD corrupted ECG signals by 3T or 7T MRI machines for accurate gating of the cardiovascular functional MRI.

## Figures and Tables

**Table 1:** Effect of various mechanisms on the performance of the proposed model.

	*3T*		*7T*	
Network	IoU (%)	DSC (%)	IoU (%)	DSC (%)
FPN	96.35	96.33	93.85	95.55
Self-FPN	97.88	98.86	95.01	97.31
Self-Attention MHDNet	98.97	99.01	97.01	98.36

**Table 2:** Performance evaluation of Self Attention MHDNet in R-peak detection for 3T and 7T settings.

Network	Magnetic Field Strength	Recall (%)	Precision (%)	F1-Score (%)
Self-Attention MHDNet	3T	99.83	99.68	99.76
	7T	99.87	99.78	99.82

**Table 3:** Comparison of this work with the current literature in R-peak detection of MHD-corrupted ECG.

Method	Magnetic Field	Precision (%)	Recall (%)	F1-Score (%)
ICA of ECG for R-peak detection [9]	7T	99.10	99.20	-
M1: R-peak detection in a single ECG lead [57]	7T	89.40	87.10	-
M2: R-peak detection in a single VCG lead [57]	7T	91.20	88.90	-
M3: 3D VCG-based R-peak detection [32]	7T	57.50	72.10	-
M5: ICA of the VCG for R-peak detection [57]	7T	87.50	84.30	-
**Self-Attention MHDNet**	**7T**	**99.87**	**99.78**	**99.82**
	**3T**	**99.83**	**99.68**	**99.76**

## Data Availability

The dataset is available in PhysioNet [48] as mentioned by the original authors in [49].
